# A new species of *Trachelas* L. Koch, 1872 (Araneae, Trachelidae) from Tajikistan

**DOI:** 10.3897/zookeys.993.59932

**Published:** 2020-11-16

**Authors:** Yuri M. Marusik, Alexander A. Fomichev

**Affiliations:** 1 Institute for Biological Problems of the North RAS, Portovaya Str. 18, Magadan 685000, Russia Institute for Biological Problems of the North RAS Magadan Russia; 2 Department of Zoology and Entomology, University of the Free State, Bloemfontein 9300, South Africa University of the Free State Bloemfontein South Africa; 3 Zoological Museum, Biodiversity Unit, University of Turku, FI-20014, Finland University of Turku Turku Finland; 4 Altai State University, Lenina Prospect, 61, Barnaul, RF-656049, Russia Altai State University Barnaul Russia

**Keywords:** Aranei, Central Asia, taxonomy, trachelids

## Abstract

A new species of trachelid spiders, *Trachelas crewsae***sp. nov.** is described from south-western Tajikistan based on both sexes. The new species is closely related to *T. vulcani* Simon, 1896 from South-East Asia but differs in the conformation of the copulatory organs and color pattern.

## Introduction

Trachelidae Simon, 1897 is a small spider group recently elevated to the family-level, consisting of 246 species in 19 genera (Ramírez 2014; WSC 2020). *Trachelas* L. Koch, 1872 is the most speciose genus of the family, accounting for 89 valid species distributed worldwide except for polar regions, Australia and New Zealand, with most of the species being known from the Americas (Platnick and Shadab 1974a, b; WSC 2020). The genus is well studied in the Palaearctic and Indomalayan regions thanks to several revisions dealing with the Mediterranean, Russian and south Chinese species (Bosselaers et al. 2009; Zhang et al. 2009; Marusik and Kovblyuk 2010; Jin et al. 2017). To date, only a single *Trachelas* species – *T. minor* O. Pickard-Cambridge, 1872, one of the most widespread species of the family – is known from Central Asia: viz., from Turkmenistan and Uzbekistan (Mikhailov 2013). While examining spiders recently collected by the senior author from Tajikistan, we found *Trachelas* specimens that belong to an undescribed species similar to the Indomalayan *T. vulcani* Simon, 1896. The goal of this paper is to provide a detailed description and diagnosis of this new species.

## Material and methods

Specimens were photographed using a Canon EOS 7D camera attached to an Olympus SZX16 stereomicroscope and a SEM JEOL JSM-5200 scanning electron microscope at the Zoological Museum, University of Turku, Finland. Photographs were taken in a dish filled with alcohol, with cotton at the bottom. The epigyne was macerated in a KOH/water solution until the soft tissues were dissolved. Digital images were prepared using Helicon Focus software (https://www.photo-soft.ru/helicon-focus/). All measurements are in millimeters. Length of leg segments were measured on their dorsal sides. Leg measurements are shown as: femur, patella, tibia, metatarsus, tarsus (total length). The terminology follows Jin et al. (2017), with some modifications. The types will be deposited in the Zoological Museum of the Moscow State University, Russia (ZMMU; curator: K.G. Mikhailov).

## Taxonomy

**Family Trachelidae Simon, 1897**

**Genus *Trachelas* L. Koch, 1872**

### Trachelas crewsae sp. nov.

Taxon classificationAnimaliaAraneaeTrachelidae

AFA28527-FC57-5EC1-9BAF-4F0797E61E25

http://zoobank.org/9FBFFDD9-4C50-420B-ADA2-D85CB1E5CF97

**Figure 1.**
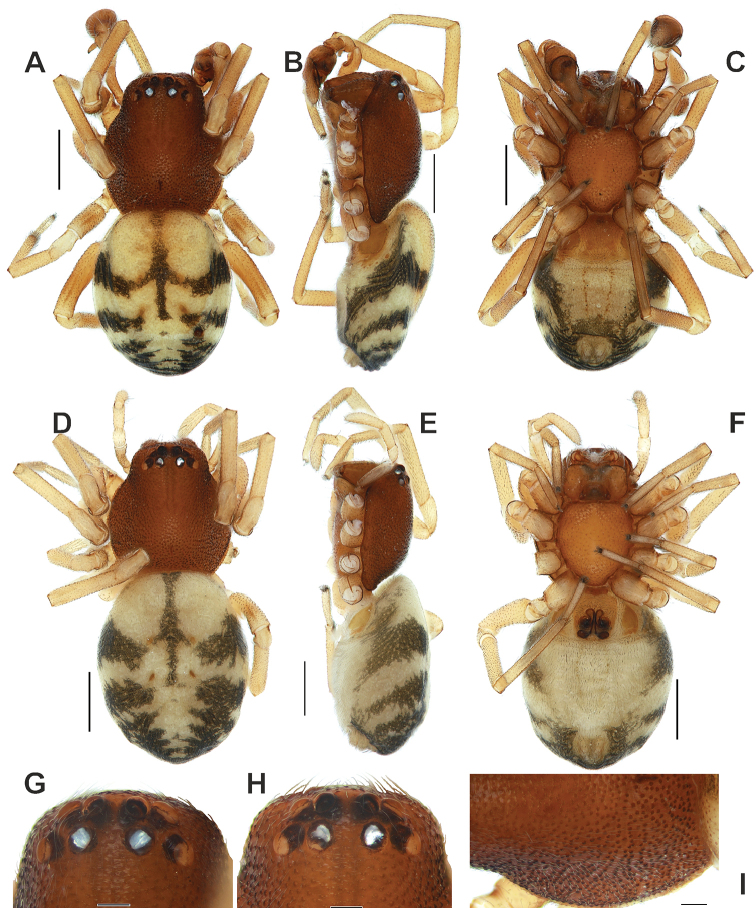
*Trachelas crewsae* sp. nov.: **A–C, G, I** male **D–F, H** female **A–F** habitus, dorsal, lateral and ventral **G–H** cephalic part, dorsal **I** carapace left side. Scale bars: 0.5 mm (**A–F**), 0.1 mm (**G–I**).

**Figure 2.**
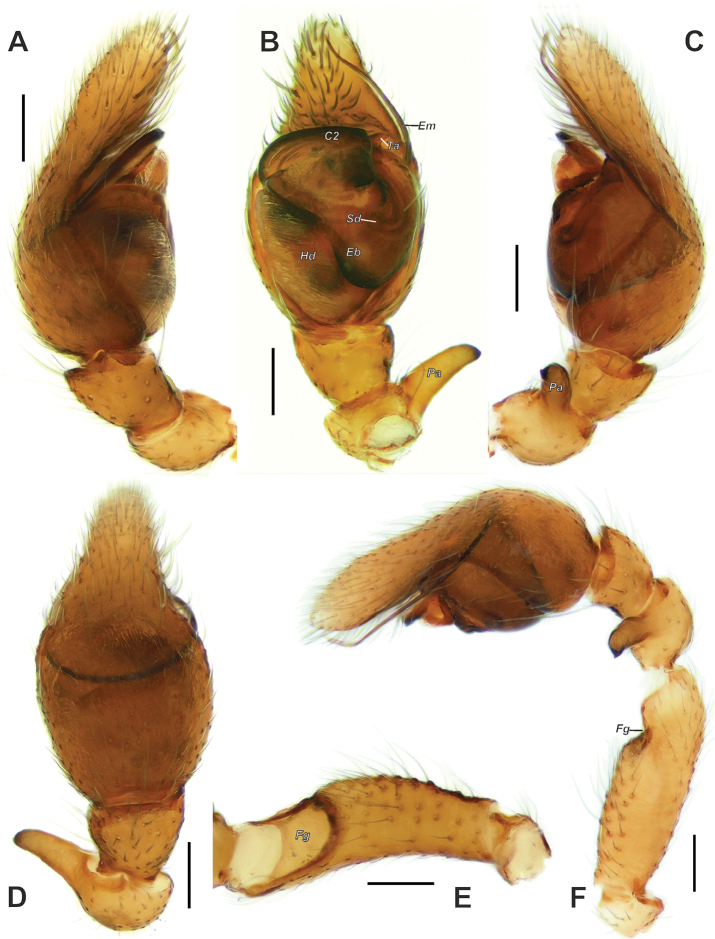
Male palp of *Trachelas crewsae* sp. nov.: **A–D** terminal part, prolateral, ventral, retrolateral, dorsal **F** whole palp, retrolateral **E** femur, ventral. Abbreviations: *C2* coil 2, *Hd* haematodocha, *Eb* embolic base, *Em* embolus, *Fg* femoral groove, *Pa* patellar apophysis, *Sd* sperm duct, *Ta* tegular apophysis. Scale bars: 0.1 mm.

**Figure 3.**
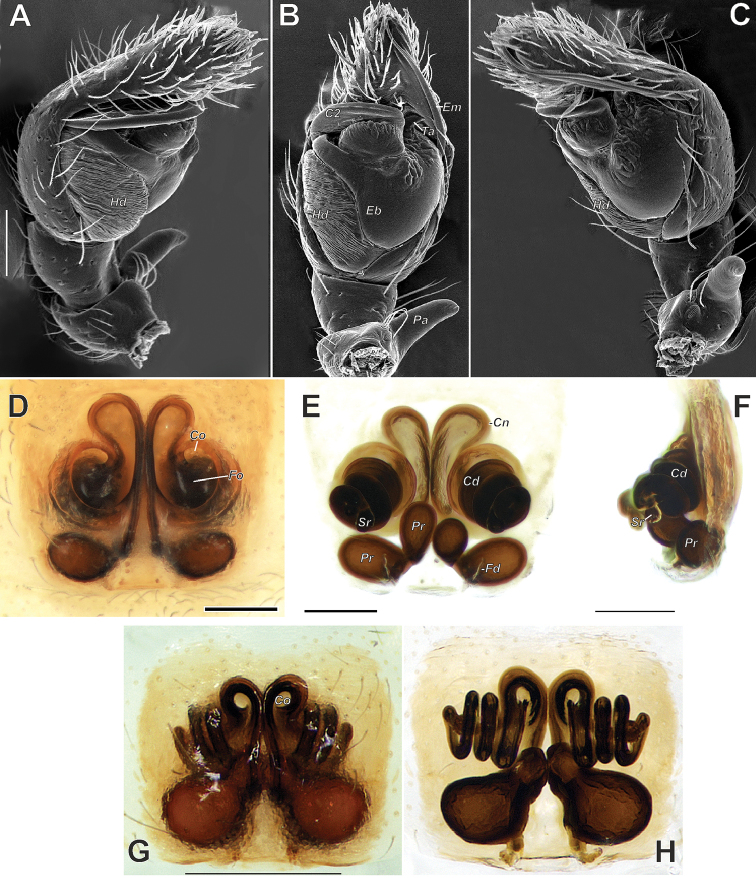
Male palp (**A–C**) and epigyne (**D–H**) of *Trachelas crewsae* sp. nov. (**A–F**) and *T. vulcani* (**G–H**) **A** prolateral **B, D, G** ventral **C** retrolateral **E, H** dorsal **F** lateral. Abbreviations: *C2* coil 2, *Cd* copulatory duct, *Cn* connecting duct, *Co* copulatory opening, *Hd* haematodocha, *Eb* embolic base, *Em* embolus, *Fd* fertilization duct, *Fo* fovea, *Pa* patellar apophysis, *Pr* primary receptacle, *Sr* secondary receptacle, *Ta* tegular apophysis. Scale bars: 0.1 mm.

**Figure 4.**
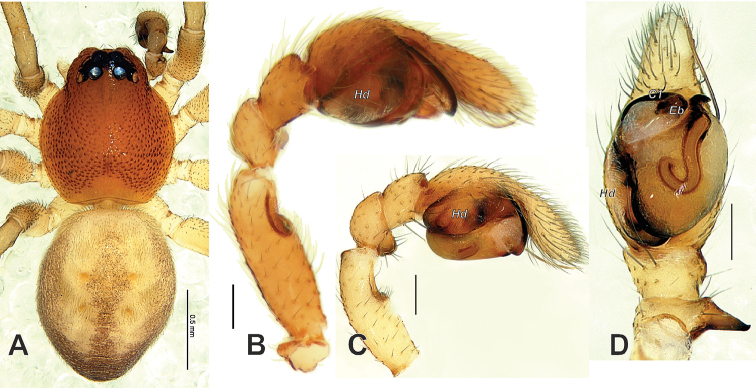
*Trachelas vulcani* (**A, C–D** from Guangxi, China) and *T. crewsae* sp. nov. (**B**) **A** male habitus, dorsal **B–C** male palp, prolateral **D** male palp, ventral. **A, C–D** courtesy of Feng Zhang. Abbreviations: *C1* coil 1 of embolus, *Eb* embolic base, *Hd* haematodocha. Scale bars: 0.5 mm (**A**), 0.1 mm (**B–D**).

**Figure 5.**
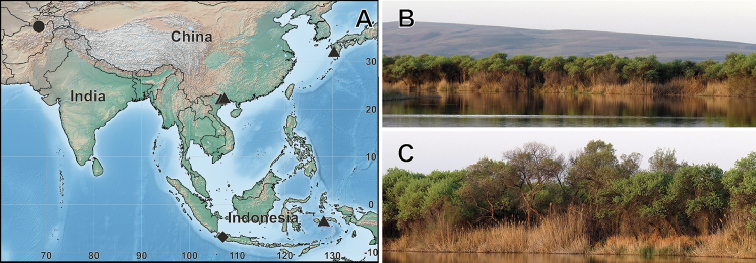
Distributional records of *Trachelas crewsae* sp. nov. and *T. vulcani* (**A**) and habitat of *T. crewsae* sp. nov. (**B–C**). Circle – *T. crewsae* sp. nov., diamond – type locality of *T. vulcani*, triangle – recent findings of *T. vulcani* outside of the type locality **B** Tigrovaya Balka Reserve **C** tugai (gallery) forest **B–C** courtesy of R.V. Yakovlev.

Figs 1 , 2 , 3A–F , 4B , 5

#### Type material.

***Holotype***: ♂ (**ZMMU**), TAJIKISTAN: Khatlon Region; Tigrovaya Balka Reserve; 37°21'20.6"N, 68°28'12.4"E; tugai (gallery) forest with thick litter; 06.05.2015 (Y.M. Marusik). ***Paratype***: 1♀ (**ZMMU**) together with the holotype.

#### Diagnosis.

The male of the new species resembles those of *T. vulcani* in having a similar long, coiled embolus and long, apically oriented patellar apophysis (*Pa*) but can be distinguished from it by having a distinct abdominal scutum occupying 2/3 of the abdomen length (vs. absent) (cf. Figs 1A, B and 4A), the patellar apophysis (*Pa*) with almost parallel edges (vs. triangular), the Ͻ-shaped sperm duct (*Sd*) (vs. J-shaped) and the haematodocha (*Hd*) being almost as wide as the tegulum in ventral view (vs. significantly narrower) (cf. Figs 2B–D, F, 3B and 4D). Males of both species are also distinguishable in the relative length/width ratio of the palpal femur (as long as cymbium in the new species vs. shorter than cymbium) (cf. Fig. 4B, C), and the much longer embolus with its base situated postero-retrolaterally vs. antero-prolaterally. The female of *T. crewsae* sp. nov. also resembles that of *T. vulcani* in having copulatory ducts packed in several coils and primary receptacles (*Pr*), consisting of two subunits, but can be separated from the latter by the copulatory openings (*Co*) situated laterally (vs. anteriorly) (cf. Fig. 3D, G), the copulatory ducts (*Cd*) packed in four tight coils (vs. three loose coils) and the secondary receptacles (*Sr*) directed posteriad (vs. anteriolaterad) (cf. Fig. 3E, F, H). Both sexes of *T. crewsae* sp. nov. differ reliably from those of *T. vulcani* in having an abdominal colour pattern formed by transverse dark grey stripes (cf. Figs 1A–F, 4A).

#### Description.

***Male*** (holotype). Total length 2.55. Carapace: 1.27 long, 1.07 wide. Carapace dark brown, granulated. Chelicerae and labium brown. Sternum yellow-orange. Maxillae light brown. Palps and legs yellow. Abdomen yellow-beige, with elongate scutum occupying 2/3 of abdomen; with dark grey dorsal pattern formed by transverse stripes; venter with epigastral scutum occupying whole ventral surface; book lung opercula large; postgaster with broad light band bordered with black lateral stripes. Spinnerets light yellow (Fig. 1A–C). Measurements of legs. I: 0.89, 0.37, 0.73, 0.56, 0.43 (2.98). II: 0.83, 0.36, 0.69, 0.53, 0.41 (2.82). III: 0.64, 0.3, 0.47, 0.5, 0.29 (2.2). IV: 0.93, 0.31, 0.79, 0.8, 0.34 (3.17).

Palp as in Figs 2A–F, 3A–C, 4B; femur as long as cymbium, three times longer than wide, with wide ventral groove (*Fg*) occupying an anterior third of segment; patellar apophysis finger-like as long as patella’s width, with a pointed tip; tegulum expanded anteriorly; Ͻ-shaped sperm duct poorly visible; embolus (*Em*) long, whip-like, coiled almost across entire tegulum; tegular apophysis (*Ta*) small, claw-shaped.

***Female***. Total length 2.7. Carapace: 1.2 long, 1.06 wide. Coloration as in the male, with lighter dorsal abdominal pattern (Fig. 1D–F). Measurements of legs: I: 0.86, 0.37, 0.67, 0.53, 0.41 (2.84). II: 0.79, 0.36, 0.64, 0.51, 0.39 (2.69). III: 0.64, 0.31, 0.47, 0.49, 0.27 (2.18). IV: 0.93, 0.33, 0.81, 0.8, 0.34 (3.21).

Epigyne as in Fig. 3D–F; epigynal plate semitransparent, through which the copulatory ducts and primary receptacles are clearly visible; fovea divided by septum ‘db’ shaped; copulatory openings small, located at anteriorly on fovea; copulatory ducts, forming four coils, packed in helix directed posteriolaterad; connecting ducts (*Cn*) looped; secondary receptacles small; primary receptacles consisting of two subunits, connected by a narrow constriction; fertilization ducts (*Fd*) weakly sclerotized.

#### Etymology.

The new species is named after our colleague Sarah C. Crews (San Francisco, USA), who continuously helps us with editing the English and providing fruitful comments on our manuscripts.

#### Distribution.

Known only from the type locality (Fig. 5A–C).

## Discussion

*Trachelas vulcani*, the sibling species of *T. crewsae* sp. nov., was described from Java, Indonesia (Simon 1896). Thereafter, the species has been recorded from Maluku Islands (Indonesia), southern China and southern Japan (Deeleman-Reinhold 2001; Jin et al. 2017; Ono and Ogata 2018) (Fig. 5A). Based on the figures from the aforementioned papers, specimens of *T. vulcani* from different localities differ in details of the male palp and epigyne. Ono and Ogata (2018) argued that these differences lie within the range of species variation, considering the wide species range. However, it is also possible that all separated populations of *T. vulcani* could belong to different, closely related species. It is necessary to re-examine the holotype of *T. vulcani* in order to resolve the matter. The present diagnosis of *T. crewsae* sp. nov. from *T. vulcani* is based on the Chinese specimens considered by Jin et al. (2017: figs 5, 6, 7, 8, 9A, B).

## Supplementary Material

XML Treatment for Trachelas crewsae

## References

[B1] Bosselaers J Urones C Barrientos JA Alberdi JM (2009) On the Mediterranean species of Trachelinae (Araneae, Corinnidae) with a revision of *Trachelas* L. Koch 1872 on the Iberian Peninsula. Journal of Arachnology 37: 15–38. 10.1636/A08-33.1

[B2] Deeleman-Reinhold CL (2001) Forest spiders of South East Asia: with a revision of the sac and ground spiders (Araneae: Clubionidae, Corinnidae, Liocranidae, Gnaphosidae, Prodidomidae and Trochanterriidae [sic]). Brill, Leiden, 591 pp.

[B3] Jin C Yin XC Zhang F (2017) Four new species of the genus *Trachelas* L. Koch, 1872 and the first record of *T. vulcani* Simon, 1896 from south-west China (Araneae: Trachelidae). Zootaxa 4324(1): 23–49. 10.11646/zootaxa.4324.1.2

[B4] Marusik YM Kovblyuk MM (2010) The spider genus *Trachelas* L. Koch, 1872 (Aranei: Corinnidae) in Russia. Arthropoda Selecta 19(1): 21–27. 10.15298/arthsel.19.1.04

[B5] Mikhailov KG (2013) The spiders (Arachnida: Aranei) of Russia and adjacent countries: a non-annotated checklist. Arthropoda Selecta 3 (Supplement): 1–262.

[B6] Ono H Ogata K (2018) Spiders of Japan: their natural history and diversity. Tokai University Press, Kanagawa, 713 pp.

[B7] Platnick NI Shadab MU (1974a) A revision of the *tranquillus* and *speciosus* groups of the spider genus *Trachelas* (Araneae, Clubionidae) in North and Central America. American Museum Novitates 2553: 1–34.

[B8] Platnick NI Shadab MU (1974b) A revision of the *bispinosus* and *bicolor* groups of the spider genus *Trachelas* (Araneae, Clubionidae) in North and Central America and the West Indies. American Museum Novitates 2560: 1–34.

[B9] Ramírez MJ (2014) The morphology and phylogeny of dionychan spiders (Araneae: Araneomorphae). Bulletin of the American Museum of Natural History 390: 1–374. 10.1206/821.1

[B10] Simon E (1896) Descriptions d’arachnides nouveaux de la famille des Clubionidae. Annales de la Société Entomologique de Belgique 40: 400–422. 10.5962/bhl.part.2026

[B11] World Spider Catalog (2020) . World Spider Catalog. Version 21.5. Natural History Museum Bern. 10.24436/2 [accessed on October 2020]

[B12] Zhang F Fu JY Zhu MS (2009) A review of the genus *Trachelas* (Araneae: Corinnidae) from China. Zootaxa 2235: 40–58. 10.11646/zootaxa.2235.1.2

